# Supplementary material for: Serum Klotho in Living Kidney Donors and Kidney Transplant Recipients: A Meta-Analysis
Source: J Clin Med. 2020 Jun 12;9(6):1834. doi: 10.3390/jcm9061834 (PMC7355868; doi:10.3390/jcm9061834)
Supplement: Supplementary file 1 [file jcm-09-01834-s001.pdf]

Supplementary Materials

Table S1. Search strategy.

Ovid MEDLINE(R) and Epub Ahead of Print, In-Process & Other Non-Indexed Citations, Daily and Versions(R)

1 klotho.mp

2 kidney transplant.mp

3 kidney transplantation.mp.

4 exp kidney transplantation/

5 renal transplant.mp.

6 renal transplantation.mp

7 exp renal transplantation/

8 kidney donor.mp.

9 2 or 3 or 4 or 5 or 6 or 7 or 8

10 1 and 9

EMBASE

('klotho protein' OR 'klotho gene') AND ('kidney transplantation' OR 'renal transplantation' OR kidney donor)

Cochrane Database of Systematic Reviews

Cochrane Reviews matching ('klotho protein' OR 'klotho gene') AND ('kidney transplantation' OR 'renal transplantation' OR ‘kidney donor’) in Title Abstract Keyword
